# Experimental and theoretical probe on mechano- and chemosensory integration in the insect antennal lobe

**DOI:** 10.3389/fphys.2022.1004124

**Published:** 2022-11-02

**Authors:** Mainak Patel, Nisha Kulkarni, Harry H. Lei, Kaitlyn Lai, Omina Nematova, Katherine Wei, Hong Lei

**Affiliations:** ^1^ Department of Mathematics, William and Mary College, Williamsburg, VA, United States; ^2^ School of Life Sciences, Arizona State University, Tempe, AZ, United States

**Keywords:** multimodal integration, antennal lobe, mechanosensory, olfactory, dynamical model

## Abstract

In nature, olfactory signals are delivered to detectors—for example, insect antennae—by means of turbulent air, which exerts concurrent chemical and mechanical stimulation on the detectors. The antennal lobe, which is traditionally viewed as a chemosensory module, sits downstream of antennal inputs. We review experimental evidence showing that, in addition to being a chemosensory structure, antennal lobe neurons also respond to mechanosensory input in the form of wind speed. Benchmarked with empirical data, we constructed a dynamical model to simulate bimodal integration in the antennal lobe, with model dynamics yielding insights such as a positive correlation between the strength of mechanical input and the capacity to follow high frequency odor pulses, an important task in tracking odor sources. Furthermore, we combine experimental and theoretical results to develop a conceptual framework for viewing the functional significance of sensory integration within the antennal lobe. We formulate the testable hypothesis that the antennal lobe alternates between two distinct dynamical regimes, one which benefits odor plume tracking and one which promotes odor discrimination. We postulate that the strength of mechanical input, which correlates with behavioral contexts such being mid-flight *versus* hovering near a flower, triggers the transition from one regime to the other.

## 1 Introduction

Olfaction is crucial for insects for a variety of behaviors, such as foraging and habitat finding. The early stage of the insect olfactory system involves hundreds of thousands of olfactory sensory neurons (OSNs) on the antennae. OSNs respond to environmental stimuli and send neural signals *via* the antennal nerve to the antennal lobe (AL), which contains thousands of projection neurons (PNs) and local neurons (LNs) segregated into clusters called glomeruli. There is generally a one-to-one mapping between OSN types and AL glomeruli—each OSN usually expresses a single type of olfactory receptor (Lancet, 1986; Chess et al., 1994; Serizawa et al., 2000) [but see (Fishilevich and Vosshall, 2005; Goldman et al., 2005; Task et al., 2022)], and the axons of OSNs expressing the same olfactory receptor tend to converge onto the same AL glomerulus (Mombaerts et al., 1996; Dynes and Ngai, 1998; Gao et al., 2000; Vosshall et al., 2000; Treloar et al., 2002). Thus, olfactory coding is high dimensional; each odor activates a subset of OSNs, leading to stimulation of the corresponding subset of AL glomeruli, while different odors activate differing (but potentially overlapping) OSN subsets (Joerges et al., 1997; Malnic et al., 1999; Wang et al., 2003). AL neurons respond to the high dimensional input coming from the antenna, while at the same time affecting one another. PNs are often connected intraglomerularly; LNs are typically connected within and across multiple glomeruli. These recurrent interactions are the first stage of significant olfactory processing, and are necessary for many critically important behavioral tasks (Martin et al., 2013). Indeed, effective olfactory processing requires coordination across the AL, as dynamical transients within the AL (spanning multiple neurons) carry more information than pooled firing rates (Christensen et al., 2003; Mazor and Laurent, 2005; Fernandez et al., 2009; Cury and Uchida, 2010).

Insects need to perform quite difficult olfactory tasks, rivaling sensory processing requirements in many vertebrates. For example, the scent of a flower is often faint and embedded within a cloud of stronger odors, and yet insects can easily identify trace amounts of a relevant odor amidst a noisy background (Saha et al., 2013; Riffell, 2014). One particularly crucial task for an insect is the ability to track an odor source mid-flight. This ability is critical for finding food or mating partners, but poses a monumental challenge: turbulent wind eddies produce complex patterns of odor strands of different sizes and concentrations intermixed with clear media, obfuscating the odor source and rarely yielding an easily discernible “concentration gradient” to follow (Murlis and Jones, 1981; Vickers, 2000; Carde and Willis, 2008) (Crimaldi et al., 2021). To successfully track an odor, an insect must simultaneously classify odor identity and rapidly resolve spatiotemporal plume dynamics mid-flight.

Evidence strongly suggests that the ability of insects to track odors mid-flight may be facilitated by the integration of mechanical input (encoding wind velocity) with chemical input (encoding odor identity and concentration). This manner of bimodality is widespread within insects (and other animals). In terms of sensory organs, while Johnston’s organ and Böhm’s bristles on the antenna are well known to detect wind velocity and guide flight maneuvers (Jarman, 2002; Sane et al., 2007; Patella and Wilson, 2018), there are also less studied bimodal sensilla (Jarman, 2002; Sane et al., 2007). For example, a subtype of trichoid sensilla on the antenna of the male hawkmoth (Lee and Strausfeld, 1990) and sensilla chaetica on the honeybee antenna (Whitehead and Larsen, 1976) exhibit chemo- and mechano-sensory bimodality. In non-insect species, such bimodality is found in sensilla on the antennules of aquatic crustaceans (Cate and Derby, 2002; Monteclaro et al., 2010; Mellon, 2012), OSNs on the septal organ and main olfactory epithelium of mice (Grosmaitre et al., 2007; Connelly et al., 2015), and mitral/tufted cells in the olfactory bulb (Iwata et al., 2017). Accordingly, a wide range of animals have been shown to be adept at intelligently sampling environmental odor plumes to home in on an odor source (Pyk, 2006; Vergassola et al., 2007; Pasternak et al., 2009; Ando and Kanzaki, 2015); moths, in particular, use a strategy in which they surge upwind upon encountering odor strands and cast across wind when losing contact with odors (Vickers and Baker, 1994). Furthermore, in honeybee hives waggle-dancers produce both vibrational and olfactory signals to transmit information about a profitable food source (Thom et al., 2007; Ai et al., 2019). This suggests that the two modalities may augment and corroborate each other, consistent with the close link between odor plume structure and air turbulence; the apparent convergence on this strategy across many species belies the importance of sensory integration in tracking odor sources.

Some of this integration of chemosensory and mechanosensory input may occur as early as at the level of the AL. There is indeed some precedent for AL bimodality—responses to both olfactory and wind stimuli have been observed in some moth AL PNs (Han et al., 2005) [and, in non-insect species, in the olfactory bulbs of tadpoles (Brinkmann and Schild, 2016) and mice (Iwata et al., 2017)]. Recently, a calcium imaging study in the honeybee AL revealed the involvement of mechanical stimulation in processing olfactory information, calling further attention to the bimodal properties of AL circuits (Tiraboschi et al., 2021).

Despite the known importance of chemical and mechanical bimodality for odor tracking, a cohesive picture of the interaction of these two information streams within the AL has remained elusive. While a robust theoretical foundation has been developed for the functional interpretation of AL olfactory responses, a similar theoretical foundation for the interpretation of AL mechanosensory responses, and the dynamical interplay between the two, is, as of yet, sorely lacking. In this review, we draw from our experimental and modeling work (Tuckman et al., 2020; Tuckman et al., 2021) to present such an overarching conceptual framework—a novel framework connecting multisensory AL dynamics with biological function and odor-seeking behavior.

Section 2 of this review discusses the anatomical substrate underlying AL bimodality, while Section 3 reviews physiological data showing bimodal interactions within the AL. Section 4 describes our modeling work incorporating multisensory integration into AL dynamics, and Section 5 develops our overarching conceptual framework for the functional interpretation of AL bimodality. Section 6 provides our concluding remarks.

## 2 Morphological connections that provide mechanosensory information to antennal lobe

### 2.1 Types of mechanical sensors on antenna

Peripheral mechanosensation includes the senses of hearing and touch, generally distinguished by their detection of vibrating waves (in air or water) or direct physical contact with external objects/substrates, respectively (Abraira and Ginty, 2013; Hudspeth, 2014). While hearing is the function of auditory organs in most vertebrates and insects, touch or tactile receptors are broadly distributed over the body, reflecting the diverse sensory functions of this modality. In the hawkmoth *Manduca sexta*, two sets of antennal mechanosensors are present on the basal antennal segments called the scape and pedicel, each set serving different functions. One set, Böhm’s bristles (Böhm, 1911), is present as fields of sensory hairs on the scape and the pedicel, serving a proprioceptive function (Krishnan et al., 2012). Roughly, these fields are opposite each other within a particular segment and orthogonal to each other across the two segments. The second set, Johnston’s Organ (Schneider, 1964), is composed of circumferentially arranged mechanosensory stretch receptors called scolopidia. Each scolopidium is innervated by a bipolar neuron. These neurons send their projections ipsilaterally *via* the antennal nerve (AN) into the antennal mechanosensory and motor center (AMMC) in the deutocerebrum of the moth (Rospars, 1988). Data from silk moths and butterflies suggest that Böhm’s bristles encode gross changes in antennal position, whereas Johnston’s Organ responds to small, high-frequency motions of the antenna, such as vibrations due to sound or air flow. Johnston’s Organ and Böhm’s bristles on insect antennae are believed to control aerial maneuvers during flight (Sane et al., 2007). In addition, hymenopterans possess Janet’s organ, a chordotonal organ that anchors within the head-scape joint (Janet, 1911). Janet’s organ detects flexion of the antennal joints in a manner not unlike the ancestral femoral chordotonal organ. Importantly, an equivalent of Janet’s organ has not yet been described in Lepidoptera and other insect orders.

In addition to Johnston’s Organ and Böhm’s bristles, there are other types of mechanosensory hairs that are distributed more broadly along the length of the flagellum, the third segment of the antenna. For example, two types of socketed mechanosensory sensilla, type-I and type-II sensillum chaetica, are distributed on the trailing edge of the flagellum of *M. sexta*. The type-I s. chaetica additionally contains chemosensory dendrites, and is therefore bimodal. These sensory axons terminate in the AL. Cobalt applied to the scratched trailing edge of the flagellum, harboring type-I and -II s. chaetica, reveals sensory endings specifically within a glomerulus’ central core (Strausfeld, 1988). This part of the glomerulus is innervated by the dendrites of a uniquely identified small-field projection neuron. Recordings from one of these cells showed it to respond to mechanosensory stimuli but not to volatiles such as female pheromones, hexanal, or amyl acetate (Kanzaki et al., 1989).

Similar mechanosensory and chemosensory sensilla are also found on the antennae of honey bees. However, these sensory hairs are located at the antennal tip, and are dubbed taste and tactile hairs (Haupt, 2007). Each taste hair houses a single mechanosensory neuron in addition to a number of chemosensory neurons, and is thus bimodal. The axons of these sensory neurons terminate at distinct regions of the dorsal lobe (DL), which is equivalent to the AMMC in moths.

Although most of the specialized mechanosensory neurons arborize in the AMMC or DL, there are several possible mechanisms that could integrate the mechano- and chemo-sensory information.

### 2.2 Possible mechanisms of bimodality

#### 2.2.1 Olfactory receptors activated directly by mechanical movement

Although experimental evidence is still lacking in insects, studies in mice have suggested that the olfactory receptor proteins may produce conformational changes upon mechanical stimulation. Patch-clamp recordings from septal organ OSNs in mice showed excitatory responses not only to odorants, but also to mechanical stimuli delivered by pressure ejections of odor-free Ringer solution. Blocking adenylyl cyclase or knocking out the cyclic nucleotide–gated channel CNGA2 eliminated both types of responses, suggesting a shared cAMP cascade underlying these responses (Grosmaitre et al., 2007). Through a series of genetic and molecular manipulations, Connelly and others showed that odorant receptors (ORs) (G-protein coupled membrane proteins on OSNs) are necessary and sufficient to produce mechanical response in the OSNs, although the response sensitivity varies among ORs (Connelly et al., 2015). The observed mechanosensitiviy could be similar to that found in another G-protein coupled receptor (NMDA) (Paoletti and Ascher, 1994). In the mammalian system, mechanosensory responses of OSNs could be used to enhance the firing frequency of individual neurons when they are weakly stimulated by odorants, and most likely drives the rhythmic activity (theta oscillations) in the olfactory bulb to synchronize with respiration.

In addition to ORs, another class of chemosensory receptors—ionotropic receptors (IRs)—is also present in the insect OSNs (Benton et al., 2009). In *D. melanogaster*, IRs are mostly housed in the coeloconic sensilla on the antennae but also widely distributed elsewhere, including the arista, pharynx, labellum, wings, legs and ovipositor (Rimal and Lee, 2018). One subclass of IRs that contains the common coreceptor *IR25a* is broadly expressed in 88% of all OSNs (Task et al., 2022), indicating their extensive participation in olfactory coding. IRs appear to be multimodal by nature, offering diverse functions such as sensation of tastes, odors, temperature and humidity (Rimal and Lee, 2018; Wicher and Miazzi, 2021). Even less explored are the incredibly diverse transient receptor potential (TRP) channels, which are involved in light sensation, thermosensation, mechanosensation and chemosensation (Fowler and Montell, 2013). Whether IRs and TRP channels are involved in mechanic sensation on antennae is still an open question, but their multimodal nature certainly invites further investigation.

#### 2.2.2 Ephaptic coupling among adjacent neurons

Distinct from chemical synapses and gap junctions, ephaptic (touching) coupling describes a form of communication among neighboring nerve cells that results from electric potentials produced by active cells propagating to nearby inactive cells through the extracellular medium. Katz and Schmitt (1940) explored the electric interaction of two adjacent limb nerves of the crab *Carcinus maenas (* *Katz and Schmitt, 1940* *),* and the term “ephapse” was suggested by Arvanitaki in 1941 for this phenomenon (Arvanitaki, 1942). Since then, the roles of ephaptic coupling have been explored in various physiological functions and clinical conditions, such as membrane excitability (Ramon and Moore, 1978), synchronization in nervous systems (Anastassiou et al., 2011) and cardiac tissue (Lin and Keener, 2010), epilepsy and seizures (Dudek et al., 1998) and olfactory coding (Bokil et al., 2001).

Within the mammalian olfactory system, the constituent fibers of the olfactory nerve are segregated into units termed fascicles, with a fascicle consisting of dozens of unmyelinated axons bundled together by a glial sheath. Within a fascicle, axons are tightly packed, with the distance between adjacent axons being in the nanometer range. The lack of myelination of individual axons and their close proximity within a fascicle create ideal conditions for intra-fascicular ephaptic communication. Indeed, through mathematical modeling, Bokil and others (Bokil et al., 2001) found that an action potential in a single axon of the olfactory nerve can evoke action potentials in all other axons within its fascicle, and also that ephaptic interactions can lead to synchronized firing of independently stimulated axons.

Within the insect olfactory system, the antennal nerve is composed of the axons of hundreds of thousands of sensory neurons distributed along the entire antenna. These axons are unmyelinated and in close spatial proximity (Banerjee et al., 2006), satisfying the conditions for producing ephaptic coupling. Additionally, on the surface of the antenna there exist various types of hair structures called sensilla, which often house dendrites from multiple receptor neurons, with some sensilla housing both chemosensory and mechanosensory receptors. Thus, these features allow the possibility of ephaptic coupling between sensory neurons of different modalities at the level of the antennal nerve (among axons) or at the sensory receptor level (among dendrites).

Ephaptic coupling may also lead to lateral inhibition. In fruit flies, activation of one OSN, either through odor stimulation or optogenetic stimulation, can inhibit a neighboring OSN in the same sensillum (Su et al., 2012). While this ephaptic coupling is bidirectional, it is not symmetric. The OSN that has the larger caliber tends to be dominant in terms of imposing inhibitory effects on adjacent OSNs (Zhang et al., 2019).

#### 2.2.3 Synaptic connections between antennal mechanosensory and motor center and antennal lobe

Interactions between sensory neurons of different modalities can also occur at the central level. In the deutocerebrum of insect brains, the AMMC or DL is the major neuropil that receives mechanosensory input, whereas the AL is the major structure that receives olfactory input. However, there exist some interneurons which connect the AMMC with the AL, providing a possible substrate for the introduction of mechanosensory input to the AL (as well as the introduction of olfactory input to the AMMC or DL).

Kymre et al. (2021) reported in the moth *Helicoverpa armigera* that there are at least two types of AL projection neurons that innervate a wide range of brain regions including the AL and AMMC. One type, named Pl_d neurons, exits the AL *via* the lateral AL tract (lALT) and innervates both the AMMC and the subesophageal zone (SEZ). Pl_d neurons are oligoglomerular with sparse branches in several glomeruli. The second type, named Pm_e neurons, shows similar connection patterns but exits the AL from the medial AL tract (mALT). The morphological features of these neurons strongly suggest that they serve multimodal roles in information processing.

In the *Drosophilla* brain, two types of AMMC neurons, AMMC-Bi16 and AMMC-Di7, innervate the AL (Matsuo et al., 2016). The AMMC-Bi16 neurons consistently innervate eight glomeruli and stochastically innervate an additional four glomeruli. The AMMC-Di7 neuron consistently innervates 3 glomeruli and stochastically innervates an additional 18 glomeruli.

In honey bees, the AMMC contains two classes of interneuron, AMMC-Int-1 and AMMC-Int-2 (Ai et al., 2017). Neither class directly innervates the AL, though AMMC-Int-2 has arborizations in the lateral horn, which connects to the AL *via* projection neurons.

## 3 Physiological evidence of bimodal responses in the antennal lobe

Experimental work both in the moth (*Manduca sexta*) and honeybee (*Apis mellifera*) have shown PN responses to nonscented air puffs, as well as elucidated the contribution of the olfactory and mechanosensory modalities to PN response patterns when the two stimulus types are presented in conjunction (Lee and Strausfeld, 1990; Han et al., 2005; Tuckman et al., 2020; Tiraboschi et al., 2021). Moreover, one particular glomerulus in the olfactory bulb (OB) of the *Xenopus laevis* tadpoles was found to respond to both chemo- and mechanosensory stimuli (Brinkmann and Schild, 2016).

In the moth, PN response patterns to mechanosensory input seem dependent on the strength of the input (Tuckman et al., 2020). Responses to high speed nonscented air puffs tend to be brief and transient, comparing with odor-evoked responses. Low-speed nonscented air puffs do not induce obvious responses, but low speed air puffs carrying odor often produce long-lasting responses. In other words, the mechanical response and olfactory response appear to operate under different time scales, with the former being more transient and the latter being longer-lasting. As may be expected from these observations, an odor stimulus delivered with high puffing speed often produces both response features in a PN: a brief, high-intensity transient response component followed by a lower-intensity, longer-lasting sustained response component. The transient component of PN odor responses at high wind speed suggests greater temporal precision, and this enhanced temporal precision makes biological sense—odor is delivered in “choppier,” briefer filaments at higher wind speed, and hence detection by the AL requires greater spatiotemporal resolution.

Mechanosensory responses in the AL were systematically studied in honey bees recently (Tiraboschi et al., 2021). Using a calcium imaging methodology, the authors demonstrated that PNs produced stronger responses to higher speed (1.8 m/s) of clean air puffs, and such responses were observed in almost all glomeruli that were accessible to imaging. Furthermore, the authors found that mechanosensory responses in PNs were diverse—some PNs were more sensitive to mechanical stimulation than others, and while most PNs produced excitatory responses to clean air flow, some actually exhibited inhibitory responses to mechanical stimuli. The wide dynamic range of these responses suggests a functional significance, and the authors indeed found an interesting possible link to behavior and intra-hive communication. The authors showed that PN mechanical responses oscillated in sync with the back-and-forth motion of an external object that mimics the natural abdominal movement of a waggle-dancing bee, with the power of these oscillatory responses peaking at the natural frequency of waggle dance motions.

In an attempt to pinpoint the source of mechanosensory input to the AL, the authors then coated the flagellum of the antenna with a layer of silicon, but left the flagellum able to freely move and bend. Two important observations were made under this treatment: 1) clean air-induced responses were completely suppressed; 2) odor could still induce responses but with an apparent delay, likely caused by slow diffusion of odor molecules through the silicon layer. These results eliminated the possibility that (in the absence of silicon treatment) responses to clean air were due to odor contamination, since (with silicon treatment) odor contaminants in the clean air stimuli should have yielded delayed responses rather than a total suppression. Furthermore, these results suggested that output from Johnston’s Organ could not be the driver of PN responses to mechanosensory input, since the coated flagellum could still move and bend and thus still activate the scolopidia of Johnston’s Organ. Thus, while these results precluded the possibility of Johnston’s Organ as a direct or indirect source of mechanical input to the AL, the other possibilities discussed in Section 2 remain viable.

In our own work, we performed extracellular recordings from the honeybee AL in order to elucidate in further detail the features of the PN mechanosensory responses that were observed in the above-mentioned calcium imaging data. In this preliminary study, we varied the air flow speed in five discrete steps in conjunction with five odor concentrations (with odor identity fixed), resulting in a set of 25 stimuli [i.e., 25 distinct (wind speed, odor concentration) pairs]. This design yielded a 5 × 5 grid of PN responses as wind speed and odor concentration are systematically varied, allowing us to use statistical analysis to disentangle the mechanical and chemical components of PN responses to odor puffs. Moreover, we measured electroantennograms (EAG), which reflect a summed potential of all activated sensory neurons on the antenna, under the same stimulus scheme. Comparison of EAG data with AL response data under identical stimulus conditions provides insight on the transformation of the sensory signal from the peripheral organ to the first synaptic center.

**FIGURE 1:**
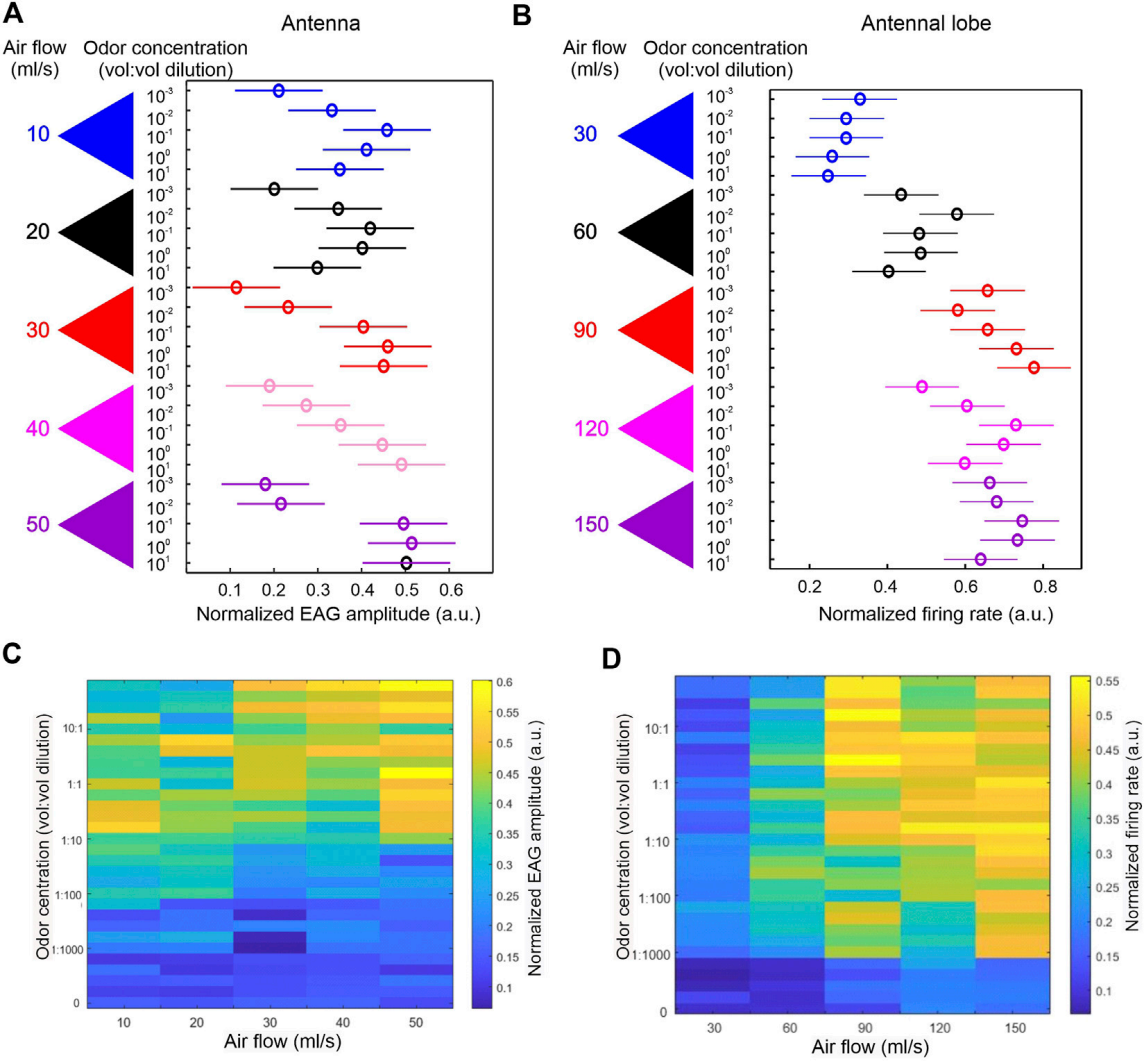
Dependency of dose responses on air flow. **(A)** Honey bees (*n* = 6) were stimulated with five concentrations of 1-Hexanol (10^–3^ to 10^1^ vol: vol dilutions) with each concentration delivered at five air flow rates (10–50 ml/s). The amplitude of electroantennogram (EAG) was measured for all combinations of concentration and air flow rate. The dose responses were significantly affected by air flow rate, as revealed by 2-way ANOVA (concentration × air flow rate: d.f. = 16, F = 2.66, *p* = 0.004). Normalized group EAG only showed linear increase with increasing concentration at intermediate air flow rates (red and pink triangles). **(B)**. Honey bees (*n* = 4) were similarly stimulated as in A but the responses were measured from AL units. 2-way ANOVA also revealed a significant interaction between concentration and air flow rate (d.f. = 16, F = 3.73, *p*<<0.0001). A linear increase of normalized firing rate following increasing concentrations was only apparent in the intermediate air flow rate (Red triangle). Experiments in **(A)** and **(B)** used different tubing systems, thus the absolute air flow readings are different. **(C)** Pseudo-colored normalized EAG response matrix showing effects of concentrations (*Y* axis) in combination with air flow rate (*X* axis). Each combination has five repeats. Concentration at zero represents non-scented air puffs. Overall the higher EAG amplitudes are distributed more towards higher concentrations. **(D)** Identical matrix using normalized firing rate from AL units. Contrary to **(C)**, stronger responses are distributed more towards higher air flow rates.

At the antennal level, both air speed and odor concentration significantly interact to modulate EAG responses (*via* 2-way ANOVA), indicating that the manner in which EAG responses vary across odor concentrations depends on the air speed. More specifically, we found that EAG amplitude increases with increasing odor concentrations—an expected dose response feature—but only at the middle range of air speeds. At low or high air speed, the positive correlation between EAG amplitude and concentration is no longer obvious (Figure 1A). A similar interaction is also apparent in AL responses (*via* 2-way ANOVA)—a positive correlation between PN response magnitude and odor concentration was mostly evident in the middle range of air speeds (Figure 1B). These results argue against the common hypothesis that olfactory responses at the antennal or AL level depend on odor flux. If this hypothesis were true, then different stimulus conditions ([wind speed, odor concentration) pairs] that maintain the same odor flux should yield similar response magnitudes; for example, an (air speed 30, concentration 10) stimulus and an (air speed 300, concentration 1) stimulus presumably result in a similar odor flux (air speed × concentration), and hence, according to the odor flux hypothesis, should yield similar response magnitudes. Thus, the odor flux hypothesis implies that, in our 5 × 5 grid of response magnitudes for varying wind speeds and odor concentrations, we should find “iso-response curves,” or regions within this matrix within which response magnitude remains constant, that occur approximately along the diagonals. However, we did *not* observe such iso-response curves within our data, and we therefore conclude that air speed does not affect response magnitudes simply by affecting odor flux, but also causes other effects—most likely mechanical responses—that are evident at both the level of the antenna and the AL.

Interestingly, we found that, while mechanical responses seem to exist at both the antennal and AL levels, the two structures seem to have different biases in integrating the two sensory modalities. At the antennal level, averaged EAG responses to all possible combinations of air speed and odor concentration (Figure 1C) show that stronger responses tend to be distributed towards higher concentrations. This is in contrast to similar data from the AL (Figure 1D), which show that stronger responses tend to be biased towards higher air speed. One possible interpretation of this result is that the stream of mechanical information is independent of the stream of chemical information, and that the relative strength of the two information streams is different at the antennal *versus* AL levels. In contrast to this interpretation, another possibility is that the streams of mechanical and odor information are identical at both the antennal and AL levels, and that the differences in response patterns at the two levels are solely due to the interplay of AL network dynamics with the incoming mechanical and chemical input. It is likely that the truth of the matter lies somewhere in between these two extremes.

**FIGURE 2:**
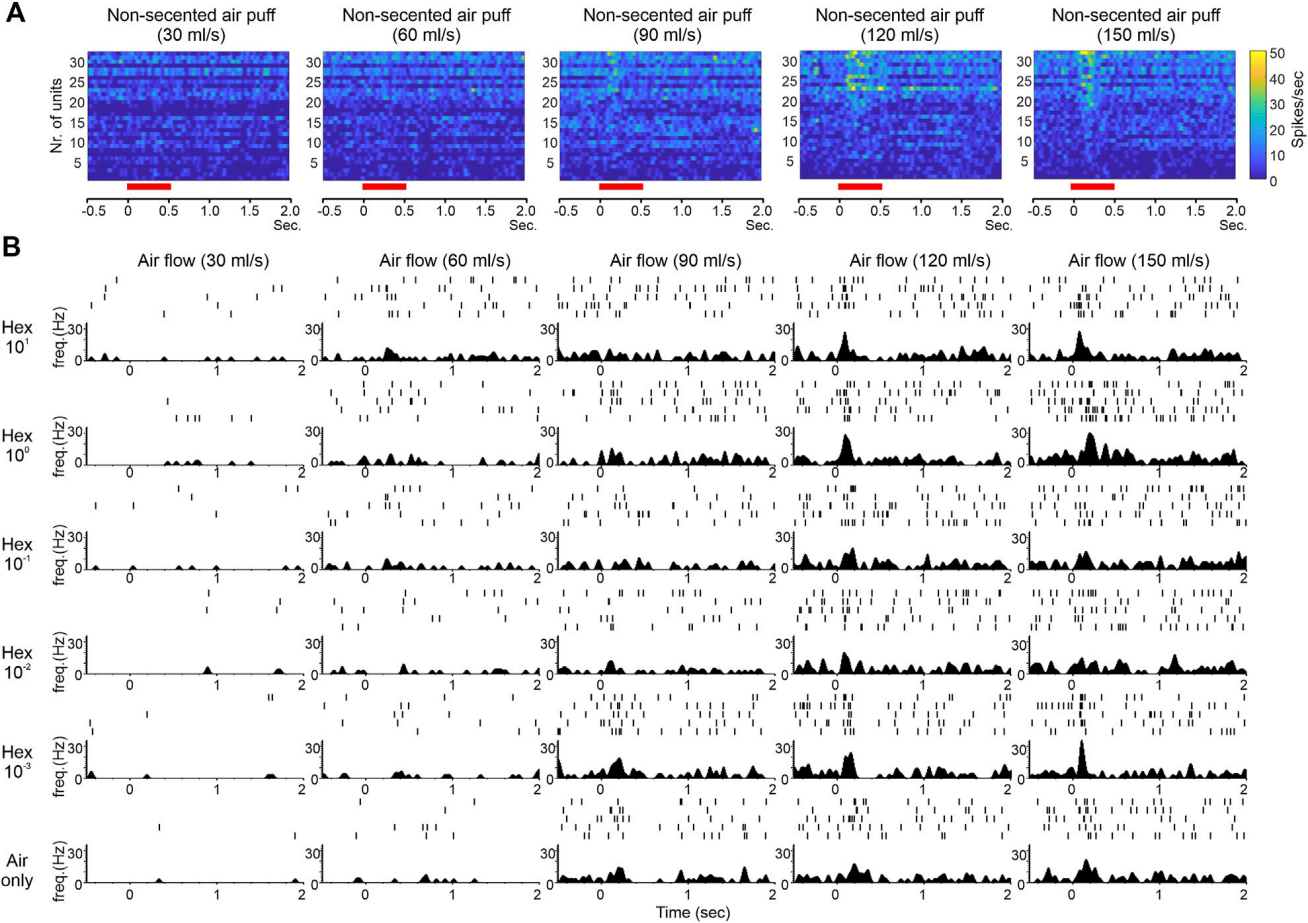
Effect of mechanical stimulation on the temporal features of odor-evoked responses. **(A)** Pseudo-color data matrices showing responses of 32 AL units from 4 bees to non-scented air puffs at different flow rate. Responses become more obvious at higher flow rates. Also apparent is the temporal transiency (more narrow in width) of the responses at the highest rate (150 ml/s). The red bars below the matrices indicate the duration of air puff. The histograms are Gaussian filtered. **(B)** Raster plots (rows of tick marks at the upper portion of each panel with each row representing one trial) and peristimulus time histograms (psth) (lower portion of each panel) showing the responses of one representative AL unit to 1-hexanol at five different concentrations (high to low from top to bottom) in conjunction of five flow rates (low to high from left to right). The bottom row shows responses to non-scented air puffs at different flow rate. Overall, the psth appears to be sharper (or more transient) at higher flow rates whether the response was elicited by odor or by air only.

Our electrophysiological recordings from the honeybee AL also allowed us to examine the detailed temporal features of bimodal PN responses. By comparing raw raster plots or raster-derived peristimulus time histograms of PN responses for different combinations of air speed and odor concentration (Figure 2), we observed: 1) clear responses to clean air puffs at high speed; 2) many responses that appeared to exhibit a transient initial peak followed by longer-lasting, lower intensity activity; 3) that the transient initial peak mentioned in (2) appeared to be associated with high air speed. These observations are consistent with our data from the moth, in which responses to odor delivered at high air speed were more transient than responses to odor delivered at low air speed (Tuckman et al., 2020). The commonality of these response dynamics among different species suggests that they serve an important biological function, likely the biological function alluded to in the beginning of this section during the discussion of our moth data (that transient, temporally precise responses at higher wind speeds allow for greater spatiotemporal resolution of the choppier, briefer odor filaments that would be present under these environmental conditions). This view is supported by our recent modeling studies, which show that mechanosensory input facilitates AL circuits in following faster stimulus pulses (Tuckman et al., 2021).

In summary, our experimental work in the moth is corroborated by the work of others and our own work in the honeybee, and the hypothesis that AL responses are bimodal is supported by an increasing amount of evidence. However, since there are no glomeruli in the AL specialized for receiving streams of mechanical information, the question of how chemical and mechanical streams of information are combined in the AL remains an open one. Further investigation is therefore still needed, and in particular studies focusing on the molecular and physiological mechanisms of AL bimodality. While continuing our query for the biological nature of olfactory bimodality, it is equally valuable to use mathematical models to address the question of how bimodal information streams interact with AL network dynamics.

## 4 Modeling work

**FIGURE 3:**
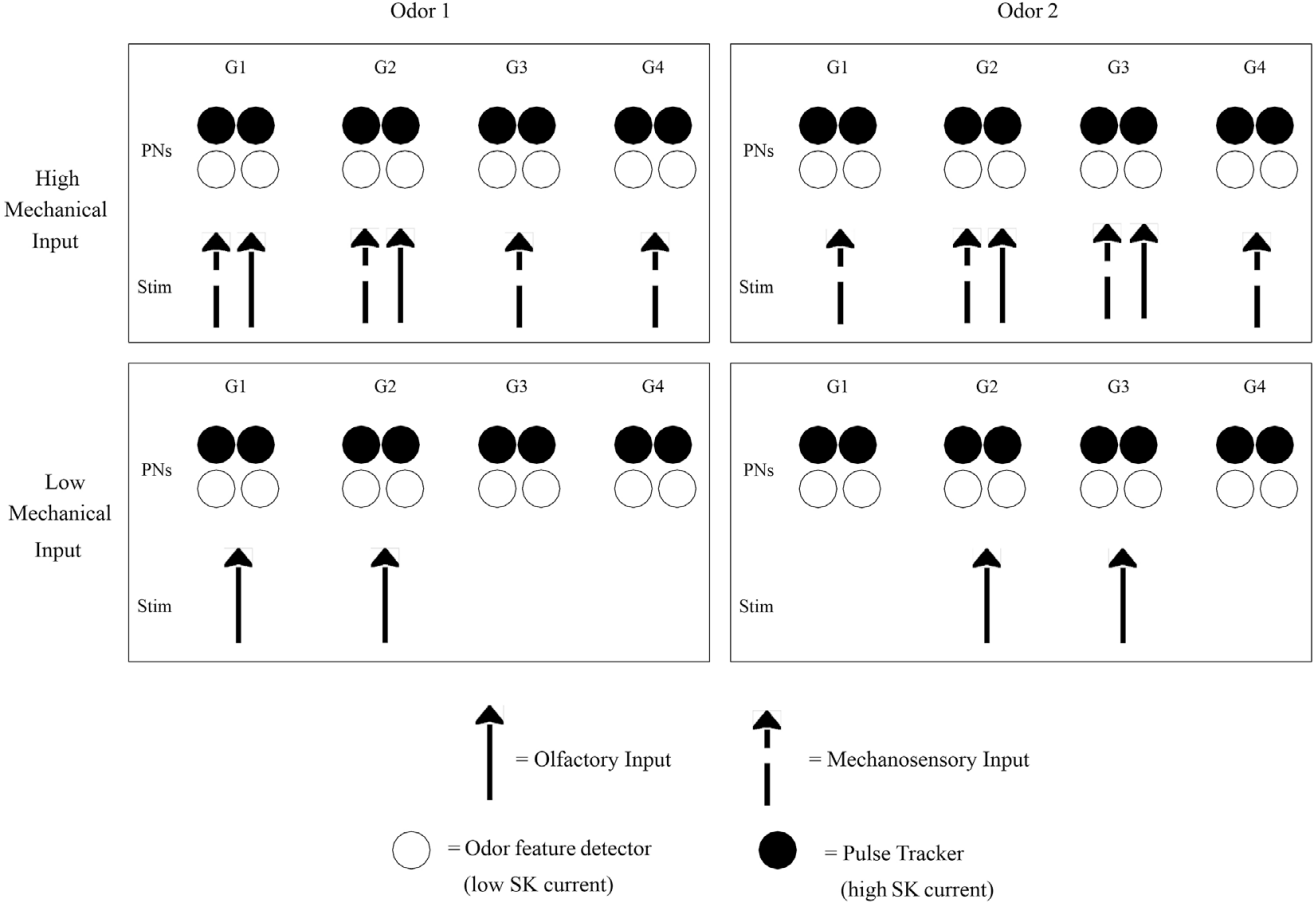
Schematic of bimodal stimulus encoding. The four glomeruli shown (G1, G2, G3, G4) each contain some PNs specialized for encoding odor features (possibly due to having weak intrinsic SK currents) and some PNs specialized for tracking the temporal dynamics of pulsatile odor encounters (possibly due to having strong SK currents). Different odors activate different glomerular subsets, while accompanying mechanosensory input has a diffuse glomerular activation pattern independent of odor identity. High Mechanical Input: This occurs, for example, during mid-flight odor-tracking, and yields the odor tracking dynamic regime. Glomeruli receiving strong olfactory input are responsible for encoding odor features, and their feature detecting PNs are the functionally relevant entities, while glomeruli receiving mechanosensory input but little olfactory input are responsible for tracking odor pulse dynamics, with their pulse tracking PNs playing the most prominent role. Since each glomerulus contains both types of PNs, every glomerulus is capable of both tasks (feature detecting and pulse tracking); the functional role a glomerulus plays depends on whether or not it is activated by the particular odor that is encountered. Low Mechanical Input: This occurs, for example, when hovering near a flower, and yields the odor discrimination dynamic regime. Pulse tracking is less important while odor discrimination is the more immediate concern, and feature detecting PNs within glomeruli activated by the encountered odor(s) play the most prominent functional role.

Our modeling work, specifically of the moth AL, is presented in detail in prior publications (Tuckman et al., 2020; Tuckman et al., 2021). We develop a biophysical spiking model of the moth AL network, consisting of PNs and LNs segregated into glomeruli, with an intrinsic calcium-dependent potassium current (the SK current) equipped to PNs and synaptic intra- and inter-glomerular fast and slow inhibition. We model both chemosensory and mechanosensory input—chemosensory input as a strong focal stimulus delivered to a subset of model glomeruli, and mechanosensory input as a weaker global signal delivered to all glomeruli (Figure 3). Thus, we capture three stimulus cases: 1) mechanosensory input alone (simulating high speed nonscented air puffs); 2) chemosensory input alone (simulating odor delivery at low wind speed); 3) chemosensory and mechanosensory input in conjunction (simulating odor delivery at high wind speed).

When employing “static” stimuli consisting of 1 s stimulus pulses (Tuckman et al., 2020), we find that, as we observed experimentally, olfactory stimulation alone leads to sustained PN responses, mechanosensory stimulation alone leads to transient responses that are sharply curtailed a few hundred milliseconds following stimulus onset, while olfactory plus mechanosensory stimulation yields higher intensity responses with both transient and sustained components. Within the model, transient responses to mechanosensory input in isolation arise primarily from the dynamics of slow inhibition coupled with the glomeruli-spanning nature of the LN network; since mechanosensory input is simulated as a weak, globally delivered signal, stimulus onset yields a burst of PN spikes across glomeruli, until (within a few hundred ms) global slow inhibition builds to sufficient strength to suppress PN responses to the weak mechanosensory stimulus. Olfactory stimulation alone, on the other hand, provides strong stimulation to only a subset of glomeruli, yielding less robust activation of the global LN network and an inability of slow inhibition to silence PN responses to the strong olfactory signal (leading to sustained responses within stimulated glomeruli throughout the duration of the stimulus). Olfactory and mechanosensory input in conjunction leads to a combination of the two effects. We note that a slow inhibitory current of the type employed in our model may be present within the ALs of other insect species as well (Barbara et al., 2005; Patel and Rangan, 2021), and these dynamics may therefore be generalizable beyond the moth. Furthermore, we find that odor classification in the model diminishes in the presence of strong mechanosensory input (due to the global, non-odor-specific nature of the mechanosensory signal), suggesting that the AL may use mechanosensory input to boost odor tracking at the cost of odor discrimination.

Static stimuli, though, fail to allow analysis of the role of mechanosensory input in odor tracking, since odor tracking entails mid-flight encounters with fragmented odor strands carried by high wind speed air pulses, and pinpointing an odor source hence requires spatiotemporal resolution of stimuli delivered in pulsatile fashion. In order to study odor tracking dynamics within our model, we constructed stimuli in the form of a series of 50 ms pulses and assessed the ability of the model AL to encode the temporal structure of pulsatile delivery (Tuckman et al., 2021). As expected from the transient nature of PN responses to mechanosensory input, we find that model PNs are better able to track stimulus pulses in the case where pulses consist of mechanosensory input alone *versus* the case where pulses consist of olfactory input alone, suggesting that the interplay of mechanosensory input with AL dynamics may indeed facilitate pulse tracking.

The more biologically relevant case, however, is that of stimulus pulses consisting of both olfactory and mechanosensory input (simulating the high wind speed odor pulses an insect is likely to encounter midflight). In this scenario, we find, in general, that the model AL is more adept at pulse tracking than in the case of olfactory stimulation alone, indicating a positive effect of mechanosensory input on odor tracking ability. Interestingly, we find a sharp discord in this case among different glomeruli; indeed, we find that PNs within glomeruli that do not receive olfactory stimulation (and receive only mechanosensory input) are highly efficacious pulse trackers (*via* their brief, transient responses), while PNs within glomeruli that receive both olfactory and mechanosensory stimulation are less effective at tracking pulses (but are more adept at encoding the length of a stimulus pulse, *via* the sustained component of their response patterns).

PNs within our model exhibit an intrinsic calcium-dependent potassium current (the SK current), which activates upon spiking and serves to reduce or suppress further spiking activity. Although SK channels are not yet found in insect AL (Bradler et al., 2016), their intrinsic properties benefit mathematical models in shaping PN’s response pattern (Belmabrouk et al., 2011). The SK current was incorporated into our model PNs too, yielding intriguing dynamical consequences. We find that, within a fixed glomerulus, variability in SK current strength among PNs not only explains the experimentally observed intraglomerular variation in PN odor response patterns (Lei et al., 2009; Lei et al., 2011), but also predicts the ability of individual PNs to track the temporal structure of pulsatile odor stimuli. The model predicts that the SK current, due to its tendency to curtail and sharpen PN responses, enhances the ability of PNs to resolve the temporal dynamics of brief odor pulses while diminishing their ability to encode the duration of more prolonged olfactory signals. Thus, within a model glomerulus, some PNs are better at encoding the temporal dynamics of pulsatile odor encounters (those with strong SK currents), while others are more adept at robustly encoding odor features, such identity or concentration, through prolonged responses (those with weak SK currents). Our work largely supports the conclusion from an independent modeling study where SK currents were found to contribute to a rapid encoding of pheromone information (Belmabrouk et al., 2011).

In addition to the intrinsic properties such as the dynamics derived from the SK channels, the neural circuitry in the AL could also affect the firing patterns of PNs. PNs are extensively modulated by LNs, most of which are GABAergic. Nagel and Wilson reported that the LNs in *Drosophila* AL exhibited diverse properties in response to pulsatile odor stimuli that emulated natural odor plumes (Nagel and Wilson, 2016). Some LNs, namely ON cells, responded transiently at the onset of stimulus; some LNs, namely OFF cells, produced longer responses after stimulus ended; yet other LNs showed both features. Although the LNs in our model do not possess the above-mentioned features, we should note that the PN intrinsic properties (e.g. SK channels) and circuit modulation are not mutually exclusive. An interesting phenomenon is that our simplified model can already capture the pulse-following feature of PNs with an acceptable confidence.

## 5 Conceptual framework

While AL bimodality has been previously documented in the literature (Han et al., 2005; Tuckman et al., 2020; Tiraboschi et al., 2021), there, as of yet, exists no broad conceptual framework for understanding the dynamical interplay of bimodal signals or the functional significance of this interplay. Our work provides a candidate for this missing framework that can guide future investigations into sensory integration within the AL.

### 5.1 Functional dynamics of bimodality

Our work is suggestive of a ‘functional division of labor’ within the AL—glomeruli that receive strong olfactory stimulation from an environmental stimulus may be responsible for encoding and conveying information about odor features such as identity and concentration (*via* their prolonged response patterns), while glomeruli that receive mechanosensory input but little olfactory input may be responsible for tracking the spatiotemporal dynamics of pulsatile odor encounters (through transient responses). Since different odors activate differing glomerular subsets, such a framework would imply that the “role” a glomerulus plays (pulse tracker *versus* feature detector) can vary from odor to odor, and hence each glomerulus must be capable of playing both roles (with environmental context determining which role is predominant).

This framework can explain the functional significance of intraglomerular PN variability; since each glomerulus must be capable of both tracking pulse dynamics and encoding odor features, it would be biologically sensible for each glomerulus to contain some PNs that are specialized for the former task while others that are specialized for the latter. Within the moth (and possibly other insect species as well), such specialization may be achieved by intraglomerular variability in the strength of the SK current equipped by PNs. When a fixed glomerulus is acting in a pulse tracking capacity (i.e., when it is only weakly stimulated by an olfactory signal), those PNs within it with strong SK currents may be more functionally relevant, while when the glomerulus is acting as a feature encoder (i.e., when it is strongly stimulated by an olfactory signal), intraglomerular PNs with weak SK currents may be the key functional entities (Figure 3).

### 5.2 Antennal lobe dynamic regimes

Furthermore, our proposed conceptual framework can be extended to connect AL dynamics with specific behavioral contexts. Insects face two intermingled tasks that are both critical for foraging success—tracking highly turbulent odor plumes and discriminating odors—and the convergence of olfactory and mechanosensory information within the AL is likely in service of these dual tasks. While, in general, both tasks must be performed concurrently, it may be advantageous for the insect to prioritize one task over the other, depending on the behavioral context. We therefore postulate that, in order to allow this context dependent prioritization, the AL may be capable of ‘transitioning’ between two distinct dynamic context-dependent regimes: an odor tracking regime and an odor discrimination regime (Figure 3).

#### 5.2.1 Odor Tracking Regime

Strong mechanosensory input, as would occur when the insect is mid-flight and actively tracking an odor source, may place the AL within the odor tracking dynamic regime. Strong mechanosensory input may “prime” the AL network, allowing an environmental odor to “push” the AL into a globally coherent state. As suggested by our experimental data, when air speed is high, strong and rapidly fluctuating mechanosensory input may induce subthreshold voltage fluctuations across PNs that are large in amplitude and tightly correlated (due to the fact that most PNs receive *the same* mechanosensory signal); embedding an odor within the windy flow, causing odor packets to “ride” atop high-speed air pulses, may then yield widespread PN spiking, tightly correlated spiking across PNs, and greater global coherence. Widespread, globally coherent AL activity may then bring full attentional resources to bear on the source-tracking task.

Furthermore, as suggested by our experimental and modeling work, strong mechanosensory input may enhance the temporal precision of PN responses, allowing the AL to rapidly resolve odor plume dynamics as the insect is attempting to pinpoint an odor source mid-flight. Glomeruli which receive strong mechanosensory stimulation, and little olfactory input, may play the most prominent role in the source-tracking task; specifically, PNs within these glomeruli that are specialized for pulse tracking, *via* their sharply transient responses, may be the key players in resolving the spatiotemporal structure of odor plumes.

On the other hand, glomeruli that receive strong olfactory stimulation may serve to signal odor identity through the longer-lasting component of their responses, and within these glomeruli, PNs specialized for feature encoding may carry the brunt of this olfactory discrimination task. We note that these PNs may have a minor role to play in source-tracking as well—if these PNs are the most adept at accurately encoding the duration of an odor pulse (as in our simulations), then they are capable of providing a rough measure of the scalar distance to the odor source, since environmental odor strands tend to become more fragmented as they meander away from their source through a turbulent medium (Yee et al., 1995; Connor et al., 2018; Pannunzi and Nowotny, 2019).

While the presence of a strong mechanosensory signal may enhance pulse tracking ability, the lack of odor specificity in the mechanical signal may impair odor discrimination, as suggested by our modeling work (Tuckman et al., 2020). Thus, the odor tracking regime may mobilize AL resources to promote pulse tracking at the cost of fine odor discrimination.

#### 5.2.2 Odor Discrimination Regime

A lack of strong mechanosensory input, as would likely occur when the insect has landed on the surface of or is hovering near an odor source (such as a flower), may place the AL within the odor discrimination dynamic regime. Near an odor source, tracking odor pulses in order to locate the source of incoming olfactory signals is less meaningful, while fine odor discrimination to parse individual odors within a cloud of olfactory signals may be a more profitable endeavor. Additionally, near an odor source, environmental odor strands tend to be larger and less fragmented than when distant from the source (Yee et al., 1995; Connor et al., 2018; Pannunzi and Nowotny, 2019), producing longer-lasting olfactory inputs that are likely to enhance odor discrimination and command corresponding behavior. Although olfactory discrimination can happen as fast as within 200 ms (Abraham et al., 2004), a longer response duration could benefit other aspects of stimulus processing, such as memory formation.

In this scenario, the absence of strong, rapidly fluctuating mechanosensory input may fail to induce the large, correlated subthreshold voltage fluctuations across AL PNs that occur in the odor tracking regime, and environmental odors may then yield less correlated and lower intensity spiking responses across PNs within responsive glomeruli. AL activity is globally unorganized, and correlations across glomeruli may be odor-dependent, with glomeruli responsive to the same component of the environmental odor blend displaying higher correlations with each other than with other glomeruli. The AL may therefore exhibit a dynamic regime in which odor-dependent local correlations, rather than global coherence, are dominant, and these “patchy” local dynamics may enhance the separation of signals from different odors.

Furthermore, PN responses in general may be more prolonged (due to longer-lasting olfactory input) and less temporally precise, with minimal activity outside of glomeruli that receive strong olfactory stimulation (due to the lack of strong mechanosensory input). As suggested by our modeling work, glomeruli receiving strong olfactory stimulation may play the most prominent role within this regime; specifically, PNs within these glomeruli that are specialized for feature detection, *via* their long-lasting responses capable of encoding the duration of an odor pulse, may be the key players in the odor discrimination task. Thus, in contrast to the globally coherent odor tracking regime, the odor discrimination regime, through its segmented dynamics, may enhance odor discrimination while sacrificing temporal precision in pulse tracking.

### 5.3 Directional and non-directional mechanosensory information

We should clarify that the mechanosensory information we are discussing is non-directional. This contrasts with the directional information encoded by dedicated mechanosensory organ such as the Johnston’s Organ (Schneider, 1964). Because there is no muscular control from pedicel to flagellum, the flagellum thus passively bends to any direction opposite to the wind. The bending direction is encoded by the sensory neurons of JO that surround the flagellum in a full circle. These neurons then transmit the information regarding the wind direction to the AMMC, which projects signal to the central body complex where the direction vectors are computed to command the corresponding turning behaviors (Matheson et al., 2022). In our model, the mechanosensory input to the AL does not carry any information about wind directions. This input only signals the intensity and frequency of mechanical stimulation. Instead of modulating the animal’s turning behavior, we hypothesize that the non-directional mechanosensory input to the AL sets a scheme for olfactory processing depending on the behavioral context.

## 6 Conclusion

We have started to see more evidence for the integration of mechano- and chemosensory information in the early processing center of the insect olfactory system, the antennal lobe. The inseparable nature of odor stimulus and air (or water) flow is reflected in a simultaneous stimulation on the peripheral organ—the pair of antennae—by wind and odor molecules. Different types of mechanical sensors on the antennae may provide mechanosensory input to the antennal lobe through several possible mechanisms, including ephaptic coupling, neuronal connections between the AMMC and the AL, or direct activation of antennal olfactory receptors by air flow (in a similar fashion to mammalian OSNs). Through modeling approaches, we suggest that the mechanical component of sensory responses within the AL may play a critical role in transitioning the AL between two distinct dynamic regimes: a fast odor tracking regime (triggered by high mechanical input) and a slower odor discrimination regime (triggered by low mechanical input). The level of mechanical input may correlate with behavioral context (e.g., high mechanical input is expected during mid-flight odor tracking, while lower mechanical input is likely when hovering near a flower), providing a link between bimodal antennal lobe dynamics and the immediate behavioral needs of the animal.

## Data Availability

The raw data supporting the conclusion of this article will be made available by the authors, without undue reservation.
