# Supplementary material for: Experimental and theoretical probe on mechano- and chemosensory integration in the insect antennal lobe
Source: Front Physiol. 2022 Nov 2;13:1004124. doi: 10.3389/fphys.2022.1004124 (PMC9667105; doi:10.3389/fphys.2022.1004124)
Supplement: Supplementary file 1 [file DataSheet1.PDF]

Supplemental methods

Honeybee preparation

Non-pollen forager bees were caught at the hive entrance each day prior to experiments. The bees were cold-anaesthetized before being harnessed on a custom-made Plexiglass block. The bee was immobilized on the block with utility wax. The bee's antennae were also immobilized at the base and the joint between flagellum and pedicel using Eicosane (a low-melting-temperature wax). For EAG recordings, the bee head was kept intact but the tip of one antenna was inserted into a glass capillary tube that was filled with 2M LiCl. For multichannel recordings, a rectangular window was cut open between the two compound eyes and between the ocelli and the bases of the two antennae. The glandular material and some trachea membranes were removed to expose the two ALs. To prevent the bee's head from pumping hemolymph and proboscis from moving, the clypeus was punctured and the muscle at the base of proboscis was severed. Eicosane was used to reseal the clypeus. The bee was positioned on a recording stage in such a way that the anterior side of the brain was facing upward. A constant saline flow was introduced into the head capsule to ensure the brain tissue was immersed in an adequate ionic environment. A standard bee saline recipe was adopted from literature (Galizia & Vetter, 2004), which contains 130 mM NaCl, 6 mM KCl, 4 mM MgCl2 x 6H2O, 5 mM CaCl2 x 2H2O, 160 mM Sucrose, 25 mM D-Glucose x H2O, 10 mM HEPES. The pH was adjusted to 6.7 and the final osmolarity was 500 mOsm. A reference electrode, made of a thin silver wire, was placed behind the brain in the saline water.

Odor delivery

Similar odor delivery systems were used in EAG and extracellular recording experiments. In brief, a charcoal filtered and moisturized air line was connected to a computer-driven solenoid valve, which controlled the air puffs going through a 1cc glass syringe that contained a piece of either odor- or solvent-laden filter paper. Care was taken to ensure that the syringes, air tubing and filter papers were clean. The speed of air flow was regulated with a Parker airflow meter. 1-Hexanol was diluted in mineral oil in a concentration series (10^{-3} to 10^{1} vol:vol dilutions). 10 µl of solution was loaded onto the filter paper in each syringe. Air puffs were directly delivered to both antennae but were closer to the ipsilateral antenna.

Stimulus panel was consisted of 5 concentrations and 5 air flow speeds, thus giving rise to 25 odor-air flow pairwise combinations. The non-scented air puff added another 5 stimuli, making a total of 30 stimuli. Each stimulus was repeated 5 times. Each stimulation lasted 0.5 sec and paused for 2 min before next stimulation. Complete set of EAG data was obtained from 6 bees and multi-channel data was obtained from 4 bees.

EAG recording

The EAG traces were acquired using an Axoclamp-2A amplifier and digitized with a National Instrument A/D board at 15 kHz. A 10x linear amplifier and low-pass filter (World Precision Instrument, Sarasota, FL) were connected to the 10x output port of the Axoclamp-2A. Data acquisition was implemented in a customized Matlab program (Lott et al., 2009) installed in a Windows PC. A typical EAG signal was a downward deflection from the baseline after the stimulus onset. The EAG amplitude was therefore

measured from the baseline to the lowest deflection point. All measurements were taken manually, aided by a customized Matlab program.

Electrophysiology in the AL

Multichannel extracellular-recording methods (Strube-Bloss et al., 2012, Lei et al., 2004, Daly et al., 2004, Christensen et al., 2000, Bennett et al., 2020) were used to measure responses of the AL.

A Tucker-Davis Technologies (TDT) RZ2 microprocessor system, in conjunction with a PZ2 preamplifier from the same manufacturer (TDT®, Alachua, FL 32615 USA) was used to digitize neural signals, which were sampled with a Neuronexus A2x2 multichannel probe (Neuronexus®, Ann Arbor, MI 48108 USA), as described in (Lei et al., 2004). Briefly, the probe was carefully inserted into the central neuropil of the AL aided with a Leica micromanipulator. After initial contact, the probe was slowly moved deeper, a few microns each step, until spikes appeared. Care was taken to insert probes approximately in the same location each time. The acquisition software was configured to acquire the spike waveforms at 25 kHz sampling rate, and the 16 recording channels were grouped in the software to form 4 tetrodes (Gray et al., 1995).

To quantify the neural responses, the tetrode waveforms, which are concatenated from four the waveforms simultaneously captured at the four contact sites within a tetrode, were exported from the TDT acquisition software to the Offline Sorter program (Plexon® Inc., Dallas, TX USA), which allows automatic as well as manual sorting of the waveforms. In Offline Sorter, each set of tetrode waveforms, composed of 32 (A/D points) x 4 (recording sites) thus 128 dimensions, was reduced to 3 dimensions using principal component analysis, and each concatenated waveform was projected onto a 3D space consisting of the 3 principal components for visualization. The quality of spike sorting was statistically verified within Offline Sorter. The time stamps of all waveforms were then exported to a spike analysis program, Neuroexplorer ® (Nex Technologies, Dallas, TX USA) or Matlab ® (Mathworks, Natick, MA USA) for further analysis.

Statistical analysis

2-way ANOVA was performed to reveal the significant interaction between odor concentration and air flow speed (Fig.1). The procedure was done within Matlab ® (Mathworks, Natick, MA USA).

Computational Modeling

The model described in this review has been explored in detail in our prior work (Tuckman et al., 2020, Tuckman et al., 2021). Model equations, parameter values, and parameter justifications can be found in the Methods sections of these prior papers. The results and conclusions from the model that are presented in this review are developed systemically and fully from model dynamics in these prior papers as well.

References

BENNETT, M. M., COOK, C. N., SMITH, B. H. & LEI, H. 2020. Early olfactory, but not gustatory processing, is affected by the selection of heritable cognitive phenotypes in honey bee. *Journal of Comparative Physiology A*.

CHRISTENSEN, T., PAWLOWSKI, V., LEI, H. & HILDEBRAND, J. 2000. Multi-unit recordings reveal context-dependent modulation of synchrony in odor-specific neural ensembles. *Nature Neurosci,* 3**,** 927-931.

DALY, K. C., CHRISTENSEN, T. A., LEI, H., SMITH, B. H. & HILDEBRAND, J. G. 2004. Learning modulates the ensemble representations for odors in primary olfactory networks. *Proceedings of the National Academy of Sciences,* 101**,** 10476-10481.

GRAY, C. M., MALDONADO, P. E., WILSON, M. & MCNAUGHTON, B. 1995. Tetrodes markedly improve the reliability and yield of multiple single-unit isolation from multi-unit recordings in cat striate cortex. *Journal of Neuroscience Methods,* 63**,** 43-54.

LEI, H., CHRISTENSEN, T. A. & HILDEBRAND, J. G. 2004. Spatial and temporal organization of ensemble representations for different odor classes in the moth antennal lobe. *Journal of Neuroscience,* 24**,** 11108-11119.

LOTT, G., JOHNSON, B., BONOW, R., LAND, B. & HOY, R. 2009. g-PRIME: A Free, Windows Based Data Acquisition and Event Analysis Software Package for Physiology in Classrooms and Research Labs". *Journal of Undergraduate Neuroscience Education,* 8(1): **A**50-A54. https://spikehound.sourceforge.net/

STRUBE-BLOSS, M. F., HERRERA-VALDEZ, M. A. & SMITH, B. H. 2012. Ensemble Response in Mushroom Body Output Neurons of the Honey Bee Outpaces Spatiotemporal Odor Processing Two Synapses Earlier in the Antennal Lobe. *PLOS ONE,* 7**,** e50322.

TUCKMAN, H., KIM, J., RANGAN, A., LEI, H. & PATEL, M. 2020. Dynamics of sensory integration of olfactory and mechanical stimuli within the response patterns of moth antennal lobe neurons. *Journal of Theoretical Biology,* 509**,** 110510.

TUCKMAN, H., PATEL, M. & LEI, H. 2021. Effects of Mechanosensory Input on the Tracking of Pulsatile Odor Stimuli by Moth Antennal Lobe Neurons. *Frontiers in Neuroscience,* 15**,** 1-17.
